# Characterization of SARS-CoV-2-specific humoral immunity and its potential applications and therapeutic prospects

**DOI:** 10.1038/s41423-021-00774-w

**Published:** 2021-10-13

**Authors:** Jiaxin Zheng, Yingying Deng, Zhenyu Zhao, Binli Mao, Mengji Lu, Yong Lin, Ailong Huang

**Affiliations:** 1 Key Laboratory of Molecular Biology of Infectious Diseases (Chinese Ministry of Education), Institute for Viral Hepatitis, Department of Infectious Diseases, The Second Affiliated Hospital, Chongqing Medical University, Chongqing, China; 2 Institute for Virology, University Hospital Essen, University of Duisburg-Essen, Essen, 45122 Germany

**Keywords:** Antibody, Humoral immunity, SARS-CoV-2, Serological antibody test, Vaccine, Infection, Antibodies

## Abstract

Coronavirus disease 2019 (COVID-19), caused by severe acute respiratory syndrome coronavirus 2 (SARS-CoV-2), is an ongoing pandemic that poses a great threat to human health worldwide. As the humoral immune response plays essential roles in disease occurrence and development, understanding the dynamics and characteristics of virus-specific humoral immunity in SARS-CoV-2-infected patients is of great importance for controlling this disease. In this review, we summarize the characteristics of the humoral immune response after SARS-CoV-2 infection and further emphasize the potential applications and therapeutic prospects of SARS-CoV-2-specific humoral immunity and the critical role of this immunity in vaccine development. Notably, serological antibody testing based on the humoral immune response can guide public health measures and control strategies; however, it is not recommended for population surveys in areas with very low prevalence. Existing evidence suggests that asymptomatic individuals have a weaker immune response to SARS-CoV-2 infection, whereas SARS-CoV-2-infected children have a more effective humoral immune response than adults. The correlations between antibody (especially neutralizing antibody) titers and protection against SARS-CoV-2 reinfection should be further examined. In addition, the emergence of cross-reactions among different coronavirus antigens in the development of screening technology and the risk of antibody-dependent enhancement related to SARS-CoV-2 vaccination should be given further attention.

## Introduction

Coronavirus disease 2019 (COVID-19) is caused by severe acute respiratory syndrome coronavirus 2 (SARS-CoV-2), and the COVID-19 pandemic has resulted in considerable morbidity and mortality [1]. The virus-specific immunity of patients with SARS-CoV-2 infection is a critical factor in the prognosis of this disease. Since the beginning of the pandemic, great progress has been achieved in gathering knowledge on the clinical characteristics of SARS-CoV-2-specific humoral immunity in COVID-19 patients. In this review, we summarize the recent progress in the characterization of the humoral immune response after SARS-CoV-2 infection. Furthermore, we discuss the significance of these findings for SARS-CoV-2 vaccine development and SARS-CoV-2-specific antibody-mediated immunotherapy.

## SARS-CoV-2 genome and lifecycle

Both SARS-CoV and SARS-CoV-2 belong to lineage B of the beta-coronavirus family [2]. SARS-CoV-2 is an enveloped, single-stranded, positive-sense RNA virus with a 30 kb genome that encodes 14 open reading frames (ORFs). Its ORFs encode four structural proteins (the spike [S], nucleocapsid [N], envelope [E], and membrane [M] proteins), 16 nonstructural proteins, and 9 putative accessory proteins (ORF3a, 3b, 6, 7a, 7b, 8, 9b, 9c, and 10) [3]. In particular, the entry of SARS-CoV-2 into host cells is mediated by the S protein through binding to angiotensin-converting enzyme 2 (ACE2), a specific viral receptor on host cells. Thus, the SARS-CoV-2 S glycoprotein is a critical target for diagnosing viral infections and developing antiviral vaccines [4]. Recent studies have shown that the SARS-CoV-2 S protein binds to the viral receptor ACE2 with a higher affinity than does the SARS-CoV S protein [5], indicating that SARS-CoV-2 has a higher interpersonal transmission rate than other emerging coronaviruses.

Upon SARS-CoV-2 infection, specific humoral immunity against this virus plays an essential role in viral clearance. Furthermore, the antibody-mediated humoral immune response plays important roles in the development and progression of COVID-19 after infection.

## SARS-CoV-2-specific humoral immunity

**Fig. 1:**
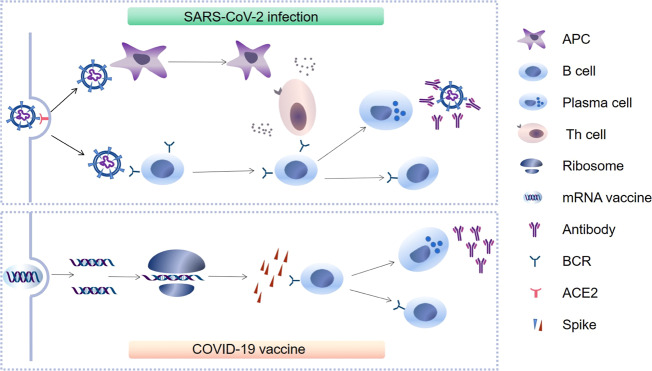
Proposed models of virus-specific humoral immunity induced by SARS-CoV-2 infection or an mRNA vaccine. (1) SARS-CoV-2 infection: SARS-CoV-2 invades host cells by both endocytosis and membrane fusion for viral entry through binding of the viral spike (S) glycoprotein to the viral receptor ACE2. Next, SARS-CoV-2 is processed by APCs, exposing its hidden epitopes. Subsequently, APCs present viral antigens to Th cells, followed by activation and lymphokine secretion and B cell activation; viral antigens may also directly stimulate B cells. Under stimulation with viral antigens, the majority of B cells proliferate and differentiate into plasma cells to produce specific antibodies against SARS-CoV-2. Simultaneously, a small proportion of B cells develop into SARS-CoV-2-specific memory B cells. Upon SARS-CoV-2 reinfection, the virus-specific memory B cells can be rapidly transformed into plasma cells to produce specific antibodies. The specific antibodies (primarily neutralizing antibodies) produced by plasma cells can neutralize SARS-CoV-2 and block the interaction between the spike protein and ACE2. (2) SARS-CoV-2 vaccine: after inoculation, an mRNA vaccine encoding the relevant S protein encapsulated by lipid nanoparticles enters a cell, and the S protein is synthesized by ribosomes. Subsequently, B cells are stimulated by the S protein to proliferate and differentiate into specific plasma and memory B cells. Finally, the plasma cells produce large amounts of specific antibodies against SARS-CoV-2, which may play a protective role in viral reinfection. ACE2 angiotensin-converting enzyme 2, APC antigen-presenting cell, BCR B cell receptor, SARS-CoV-2 severe acute respiratory syndrome coronavirus 2, Th cells T helper cells

When foreign pathogens invade the human body, the immune system initiates and induces a cascade of immune responses to clear the pathogens. Adaptive immunity, including humoral and T cell-mediated immunity, plays a critical role in the elimination of pathogens, including SARS-CoV-2. Cytotoxic lymphocytes (primarily cytotoxic CD8^+^ T cells) can eliminate infected cells, and specific antibodies against SARS-CoV-2 in the humoral immune response have the potential to neutralize this virus or even help cytotoxic T cells eliminate virus-infected cells to control disease progression [6]. Under stimulation by SARS-CoV-2 antigens, B cells from germinal centers can proliferate and differentiate into plasma cells, producing and secreting specific antibodies to control viral replication (Fig. 1). Virions may also directly modulate host-specific immunity by infecting immune cells expressing the viral receptor ACE2, such as pulmonary monocytes and macrophages. These antibodies can be present in the blood or produced de novo by memory B cells and plasma cells upon re-exposure to viral antigens [7]. Thus, SARS-CoV-2-specific humoral immunity plays a critical role in antiviral defense by providing newly produced antibodies from activated plasma cells.

### Pre-existing SARS-CoV-2-specific humoral immunity

SARS-CoV-2, SARS-CoV, and Middle East respiratory syndrome coronavirus (MERS-CoV) belong to the same class of emerging coronaviruses that include positive-sense, single-stranded RNA viruses and share a certain percentage of genetic similarities [8]. Thus, a certain amount of immune cross-reaction may exist among these three emerging coronaviruses, which may provide temporary and adequate immune protection against different coronaviruses. Relevant studies have shown that there may be a pre-existing antibody recognizing SARS-CoV-2 in uninfected individuals, particularly in children and adolescents [9]. Therefore, identifying the conserved epitopes in S2 by targeting neutralizing antibodies (NAbs) may aid the development of a universal vaccine that protects against both current and future coronaviruses.

### Methods for evaluating SARS-CoV-2-specific humoral immunity

Based on the SARS-CoV-2 genome, nucleic acid tests using multiplex polymerase chain reaction (PCR) detection technology have been implemented for first-line diagnosis of COVID-19 patients globally [10]. With the widespread use of reverse transcription PCR (RT–PCR), the limitations of nucleic acid tests inevitably appear, particularly in the cases of asymptomatic infections and herd immunity assessments [11]. Serological antibody tests, such as tests detecting IgA, IgM, IgG, or total antibodies specific for different SARS-CoV-2 antigens (including the full-length S protein, spike receptor-binding domain (RBD) of S, and N protein), are urgently required to complement nucleic acid tests. To help diagnose SARS-CoV-2 infection, many laboratories and pharmaceutical companies are aiming to manufacture various serological antibody test kits with high specificity and sensitivity, including enzyme-linked immunosorbent assay (ELISA), chemiluminescent immunoassay (CLIA), chemiluminescence microparticle immunoassay (CMIA), lateral flow immunoassay (LFIA), and magnetic chemiluminescence enzyme immune-assay (MCLIA) kits [12–15]. In addition, recent studies have explored related methods, using flow cytometry or the commercial enzyme-linked immunospot (ELISpot) assay, for detecting SARS-CoV-2-specific B cells, which produce antibodies that provide protective immunity [16–18]. Therefore, SARS-CoV-2-specific antibody tests and B-cell measurements are required to characterize specific humoral immunity in COVID-19 patients.

## Characterization of SARS-CoV-2-specific humoral immunity in COVID-19 patients

To investigate the dynamic characteristics of humoral immunity during SARS-CoV-2 infection, many studies have focused on evaluating the clinical characteristics of antibody responses in COVID-19 patients [14, 15, 19–23]. COVID-19-recovered subjects produce IgG antibodies targeting viral S and N proteins and the RBD, which are of particular relevance for their high likelihood for a neutralizing capacity [24]. The levels of SARS-CoV-2 N- and S-specific IgM and IgG antibodies in COVID-19 patients are related to serological diagnosis and prognosis prediction [25]. However, whether qualitatively different antibodies evolve in individuals with severe infections and whether antibody function changes with disease severity remain unclear. Understanding how antibody function evolves during infection and how to promote recovery rather than pathology may provide critical insights for vaccine and treatment design to avoid potential harm and provide efficient humoral defenses [26]. Many studies have evaluated SARS-CoV-2-specific IgM, IgG, and IgA antibodies in COVID-19 patients at various disease stages and degrees of severity and analyzed the characteristics of the specific antibodies [27–29]. We have summarized the findings of related studies characterizing SARS-CoV-2-specific humoral immunity in COVID-19 patients (Table 1).

**Table 1:** Characteristics of specific antibody response against SARS-CoV-2

COVID-19 patients	Positive rate (%)			Peak time (days)			Median seroconversion time (days)			Method	References
	IgM	IgG	IgA	IgM	IgG	IgA	IgM	IgG	IgA		
140	92.7	77.9	–	15–21	–	8–14	5	14	–	ELISA	[8]
80	93.8	93.8	–	–	–	–	18	20	–	ELISA, LFIA, CMIA	[12]
79	>95.0	>95.0	–	>20	>20	–	–	–	–	ELISA, LFIA	[14]
173	–	>99.0	–	–	–	21	–	–	–	ELISA	[15]
76	–	>90.0	–	16–20	16–20	–	–	–	–	CLIA	[19]
85	100.0	95.0	–	–	–	–	–	–	–	ELISA, LFIA	[20]
87	96.8	96.8	98.6	11–15	31–41	16–20	4–6	5–10	4–6	CLIA	[21]
229	–	–	–	7–10	–	–	–	–	–	ELISA	[23]
173	98.6	99.0	–	–	–	–	12	14	–	ELISA	[29]
285	94.1	100.0	–	20–22	17–19	–	13	13	–	MCLIA	[30]
94	84.6	80.8	–	–	–	–	8	20	–	–	[33]
37	93. 4	95.1	98.9	–	–	–	14	14	13	CLIA	[36]

*MCLIA* magnetic chemiluminescence enzyme immune-assay, *ELISA* enzyme-linked immunosorbent assay, *CMIA* chemiluminescence microparticle immunoassay, *CLIA*, chemiluminescent immunoassay, *LFIA* lateral flow immunoassay

### Dynamic characteristics of the SARS-CoV-2-specific antibody response

Most COVID-19 patients produce one or more specific antibodies against SARS-CoV-2. IgM and IgG antibodies have always been the focus when describing immune characteristics, whereas mucosal and systemic IgA reactions, which may also play an important role in the pathogenesis of this disease, have received less attention. To date, some studies have revealed several types of seroconversion after SARS-CoV-2 infection. We previously reported acute antibody responses to SARS-CoV-2 infection in 285 COVID-19 patients [30]. IgM and IgG titers tended to be stable within 6 days after seroconversion, and all these patients achieved IgM or IgG seroconversion within 20 days of symptom onset. The proportion of patients positive for virus-specific IgG was approximately 100% within 17–19 days of symptom onset, while that for IgM peaked at 94.1% approximately 20–22 days after symptom onset. The median time of both IgG seroconversion and IgM seroconversion was 13 days after symptom onset. Furthermore, we observed three types of seroconversion: synchronous IgG and IgM seroconversion (nine patients), IgM seroconversion that occurred earlier than IgG seroconversion (seven patients), and IgM seroconversion that occurred later than IgG seroconversion (ten patients). Subsequently, Lou et al. also described antibody dynamics in 80 COVID-19 patients [12]. They found that the IgM and IgG seroconversion rates were both 93.8%. The median seroconversion times for IgM and IgG were 10 and 12 days after symptom onset, respectively. Moreover, in a study of 173 patients by Zhao et al., the positive seroconversion rates for IgM and IgG were 82.7% and 64.7%, respectively. The mean seroconversion times for IgM and IgG were 12 and 14 days, respectively. The positive antibody rate was <40% within 1 week of symptom onset and increased rapidly to 100.0% (IgM or IgG), 94.3% (IgM), or 79.8% (IgG) after 15 days [29]. Furthermore, in a cohort analysis including 94 COVID-19 patients, 56 (59.6%) and 46 (48.9%) patients exhibited detectable IgM and IgG antibodies, with the median seroconversion times for IgM and IgG being 10 and 12 days, respectively [31]. The peak value of specific antibodies within 6–20 days was significantly higher in patients with severe disease than in those with mild symptoms. Thus, there are several types of seroconversion in COVID-19 patients as well as different kinetics of IgM and IgG antibodies specific for SARS-COV-2.

Notably, there are limited studies analyzing the levels of IgA in COVID-19 patients. An important study from Ma et al. was performed to analyze the levels of SARS-CoV-2 RBD-specific IgM, IgG, and IgA antibodies in 87 COVID-19 patients [21]. Consistent with the studies mentioned above, viral RBD-specific IgM antibodies provide better diagnostic outcomes at early disease stages, whereas IgG antibodies are preferred at later disease stages. Ma et al. further found that the median levels of RBD-specific IgA began to decline after the peak during 16–20 days post illness onset but remained at relatively high levels until 31–41 days, indicating that IgA is powerful for diagnostics at both early stages and later stages. Subsequently, Cervia et al. further revealed that a significant increase in SARS-CoV-2 S protein-specific serum IgG and IgA antibody production develops in severe COVID-19 patients after symptom onset, with very high serum IgA titers correlated with severe acute respiratory distress syndrome [15]. However, mild COVID-19 is associated with sustained secretion of mucosal SARS-CoV-2 S protein-specific IgA but transient production of serum IgG and IgA antibodies. Overall, further studies on the kinetics and mechanism of the antibody response against SARS-CoV-2 are warranted, particularly on systemic and mucosal SARS-CoV-2-specific IgA in a large population of COVID-19 patients.

### Correlations of anti-SARS-COV-2 antibody levels with COVID-19 disease severity and course

The levels of serum SARS-CoV-2-specific IgM, IgG, and IgA antibodies reflect clinical disease severity and disease-resolution outcome, and these associations are beneficial for informing therapeutics for COVID-19 treatment. A retrospective analysis of 192 patients revealed that the IgM level in patients with mild disease was significantly different from that in patients with severe disease [32]. In particular, the IgM titer of patients with severe disease was significantly higher than that of patients with mild disease on day 6 after symptom onset. Furthermore, the total antibody titer of patients with severe disease was significantly higher than that of patients with mild disease from day 7 to day 42 after symptom onset. A cohort study by Lynch et al. revealed that the peak value for IgM antibodies in ICU patients was significantly higher than that in patients with mild disease between 6 and 20 days, whereas the peak value for IgG antibodies was significantly higher in ICU patients than in patients with mild disease at all time intervals beginning 5 days after symptom onset [33]. Wang et al. revealed that elevated levels of serum IgM antibodies and NAbs in COVID-19 patients were associated with a poor prognosis [34]. In addition, Casadevall et al. revealed that higher levels of SARS-CoV-2 S protein-specific antibodies were produced in severe patients than in mild patients and the antibody titers persisted longer in severe patients than in mild patients [35]. All the mentioned studies revealed that cell-mediated and humoral adaptive immune responses may be stronger in symptomatic patients than in asymptomatic patients, indicating that uncontrolled adaptive responses may be involved in the secondary phase of immunopathology.

Recent studies support that sustained high plasma levels of SARS-CoV-2-specific IgA are associated with severe progression and a poor disease-resolution outcome [15, 21, 23, 36]. An important study, which included 37 COVID-19 patients, reported that the levels of virus-specific IgG and IgA antibodies in severe patients were significantly higher than those in nonsevere patients, whereas there was no significant difference in IgM levels [36]. The study further revealed a significant positive correlation between the levels of SARS-CoV-2 S protein-specific IgA and the Acute Physiology and Chronic Health Evaluation-II score in severe COVID-19 patients. Moreover, Cervia et al. observed that higher levels of SARS-CoV-2 S protein-specific serum IgG and IgA antibodies existed in severe COVID-19 patients than in patients with mild disease and that high levels of serum IgA antibody were significantly correlated with severe acute respiratory distress syndrome [15]. In addition, another two independent studies by Ma et al. and Tang et al. suggested that elevated IgA antibody levels were associated with severe COVID-19 prognosis [21, 23]. Collectively, serum SARS-CoV-2-specific antibody levels (particularly IgA) indicate the disease severity and disease-resolution outcome, but the definite molecular mechanism needs to be further investigated.

### Differences in SARS-CoV-2-specific humoral immunity between asymptomatic and symptomatic patients

We previously examined 37 asymptomatic individuals in Wanzhou, Chongqing, China who were diagnosed with RT-PCR-confirmed SARS-CoV-2 infection but without any relevant clinical symptoms in the preceding 14 days and during hospitalization [37]. The asymptomatic group had a significantly longer viral shedding duration than the symptomatic group but lower levels of virus-specific IgG and 18 pro- and anti-inflammatory cytokines. Subsequently, our study revealed that the proportion of SARS-CoV-2 RBD-specific memory B cells that persisted in the peripheral blood of individuals who recovered from symptomatic infection was significantly higher than that in individuals who recovered from asymptomatic infection and healthy controls [38]. This indicates that low frequencies of virus-specific memory B cells may persist in individuals who recover from asymptomatic SARS-CoV-2 infection. Moreover, a retrospective study of 37 cases revealed significantly higher serum levels of SARS-CoV-2-specific IgM, IgG, and IgA antibodies in the symptomatic group than in the asymptomatic group [39]. Li et al. found that long-term viral shedding was observed in patients with mild symptoms and asymptomatic patients [40]. Using serum proteome microarray analysis, they further demonstrated that of 20 SARS-CoV-2 proteins, the asymptomatic patients primarily produced IgM and IgG antibodies against the S1 and N proteins. In addition, a study from South Korea reported that the humoral immune function of COVID-19 patients gradually weakened and that the NAb titer decreased more in symptomatic patients than in asymptomatic patients [41], reinforcing the concern that the specific humoral immunity acquired following natural SARS-CoV-2 infection may not last long. Overall, the existing evidence suggests that asymptomatic individuals have a weaker immune response and memory B cells following SARS-CoV-2 infection than symptomatic individuals.

### Characterization of SARS-CoV-2-specific humoral immunity in pediatric COVID-19 patients

Although the epidemiological and clinical characteristics of pediatric COVID-19 patients have been reported previously, there is limited information on the characteristics of SARS-CoV-2-specific humoral immunity. Previous studies have reported that protective humoral immunity is observed in SARS-CoV-2-infected children, including the presence of specific antibodies against SARS-CoV-2 and specific memory B cells [42]. Moreover, the B-cell immune response in virus-infected children was more active than that in uninfected children within 2–3 weeks of disease onset. This effective humoral immune response may explain why most SARS-CoV-2-infected children recover more easily than adults. Furthermore, a study by Wu et al. demonstrated that the number of lymphocytes (including T and B cells) was increased; however, the number of neutrophils was decreased during COVID-19 progression in pediatric patients. The titers and duration of IgG antibodies against SARS-CoV-2 in pediatric patients exhibited no significant differences compared with those in adult COVID-19 patients [43]. Moreover, recent serological studies have shown that asymptomatic or subclinical infections and transmission through children are rare [44]. Notably, a survey of COVID-19 patients in New York revealed that the titer of serum NAbs and antibody-dependent cellular phagocytosis in adults were higher than those in children. Furthermore, NAb titers were positively correlated with age [45]. These results also indicate that the poor prognosis of adults cannot be simply attributed to a failure to produce an antibody-mediated immune response. Owing to the influences of social factors and age structure, the differences in infection rates and humoral immunity in children need to be further investigated.

## Potential application and therapeutic prospects of SARS-CoV-2-specific humoral immunity

### Assistive diagnostic tool for SARS-COV-2 infection

As SARS-CoV-2 is a novel emerging virus, its characteristics and detailed mechanisms related to humoral immunity in COVID-19 patients are not well defined. RT-qPCR is the gold standard diagnostic technique because of its sensitivity and specificity [46, 47]. However, false-negative findings occur in ~30% of COVID-19 patients, which may have severe consequences, as contagious individuals will inevitably spread the disease [8]. Although RT-qPCR remains the first choice for the diagnosis of COVID-19, serological antibody tests are an assistive technique that can help us understand the characteristics of the SARS-CoV-2-induced immune response and identify asymptomatic individuals with negative RT-PCR results. Our previous study revealed that the detection of virus-specific antibodies for COVID-19 could be used as an assistive technique for the diagnosis of suspected cases with negative RT-PCR results and for surveying asymptomatic infection in close contacts [30]. In our small-scale investigation, seven asymptomatic patients with negative nucleic acid results were positive for IgG and/or IgM. This study highlights the importance of serological testing for a more accurate estimation of COVID-19 prevalence.

Serological antibody detection can reflect the natural history of infection and accurately determine transmission and mortality rates. Therefore, serological detection is an indispensable tool in the management of infectious diseases, including their diagnosis, determination of the protective antibody titer after vaccination, and epidemiological evaluation of humoral immunity [48].

### Serological surveys contribute to epidemiological management

Owing to the global SARS-CoV-2 outbreak, population-based data on COVID-19 are essential for guiding and responding to health safety policies. However, owing to the limitations of detection methods, the existence of asymptomatic infections, and incomplete reporting of cases to public health authorities, there are still many unreported cases. As antibody prevalence can reflect the cumulative infection rate, a SARS-CoV-2-specific seroprevalence survey can provide supplementary information for considerable geographic, community-level, and detailed population research. In the context of the SARS-CoV-2 outbreak, serological surveys are one of the best tools for determining the spread of this infectious disease. This analysis is helpful in the evaluation of changes in specific antibody levels after SARS-CoV-2 infection and the relationships between these changes and related factors, such as sex, age, existing phenotype, and SARS-CoV-2 symptoms.

However, limited studies have investigated seroprevalence in certain areas, particularly in developing countries [49]. In a recent study, we clearly described the entire outbreak of SARS-CoV-2 transmission in Wanzhou, Chongqing, China [50]. We found that asymptomatic and presymptomatic transmission in the general population accounted for 75.9% of the next generation of infections, which was due to transmission from close contacts before symptom appearance or diagnosis in 80.7% of symptomatic cases and 19.3% of asymptomatic cases. We further evaluated the effectiveness of control measures by analyzing the transmission of asymptomatic and symptomatic cases in each generation and the exposure patterns that may have facilitated transmission in the study population during the outbreak. Moreover, a recent study revealed that the infection rate was 0.9% and the infection fatality risk was 0.3% during the first wave of the COVID-19 pandemic in Iceland. Moreover, 56% of infected people were diagnosed using RT-qPCR, 14% of whom were isolated, and 30% neither knew that their RT-qPCR results were positive nor were isolated [51]. Therefore, despite extensive screening using RT-qPCR, a substantial proportion of infected individuals were unaware of their infected status, indicating that there is a fair proportion of infected individuals without substantial symptoms. Stringhini et al. conducted a weekly survey of the seroprevalence of anti-SARS-CoV-2 antibodies in the population of Geneva, Switzerland [52]. Their retrospective analysis revealed that the seroprevalences in children aged 5–9 years and adults over 65 years were significantly lower than that of individuals aged 10–64 years, providing valuable information to the government for focusing on protecting the vulnerable population. A population-based serological epidemiological study conducted in Spain revealed that the prevalence of SARS-CoV-2 IgG antibodies was ~5% [53]. In this study, significant regional differences were observed between the central and suburban areas of Spain. This study obtained representative national and provincial data that can provide reference data for epidemic prevention and control.

Overall, serological antibody investigation is of great significance for assessing the transmission characteristics of SARS-CoV-2 in different countries and regions. Regardless of whether humoral immunity is sufficient to protect against reinfection, low SARS-CoV-2-specific antibody seroprevalence indicates that populations are vulnerable to a second wave of infection.

### Development of antibody-mediated immunotherapy against SARS-COV-2 infection

Effective humoral immunity is essential for eliminating viruses and preventing viral reinfection. Specific antibody responses induced by SARS-CoV-2 infection may help restrain the infection and spread of the virus in the human body via NAb production [54]. Thus, the development of specific antibody-mediated strategies may be critical for prophylaxis, postexposure prophylaxis, or treatment of SARS-CoV-2 infection. Many NAbs against SARS-CoV-2 block the binding of the RBD and ACE2 by targeting the RBD of the SARS-CoV-2 S protein [55, 56]. Zost et al. revealed that monoclonal NAbs isolated from patients with severe COVID-19 can neutralize SARS-CoV-2 in vitro [57], indicating that specific humoral immunity can effectively eliminate the virus. In addition, Chi et al. revealed that naturally occurring human SARS-CoV-2-specific monoclonal antibodies isolated from the B cells of 10 recovered donors differed in gene usage and epitope recognition of the viral S protein. These unexpected results indicate that there are other important mechanisms involved in the neutralization of SARS-CoV-2, in addition to inhibiting the interaction between the virus and its receptor [58]. Furthermore, specificity and kinetic analyses of NAbs induced by SARS-CoV-2 infection are vital for understanding immune protection and determining vaccine design goals. Therefore, these antibodies are of great significance for the prevention of viral infections and are useful tools for the development of effective treatment methods [59].

However, whether the produced NAbs are sufficient to combat SARS-CoV-2 remains controversial. Little is known about the longitudinal dynamics of SARS-CoV-2-specific NAbs in COVID-19 patients [60]. Antibodies against the SARS-CoV-2 S protein seem to protect against lethal coronavirus attacks and clear viruses from mice and ferrets [61, 62]. A study of the immune response in rhesus monkeys revealed that the monkeys developed diseases similar to those of humans when exposed to SARS-CoV-2. Notably, SARS-CoV-2 infection could partly protect the macaques from a second attack by the virus because of the production of protective antibodies [63, 64]. Although studies have confirmed that NAbs can help the body effectively eliminate a virus, the results of human clinical trials are crucial. Previous studies have revealed that the timing and quality of SARS-CoV-2-specific antibody responses can provide a reasonable basis for determining when exogenous antibody administration to COVID-19 patients would have the greatest beneficial effect [35, 65, 66].

Plasma therapy is based on the use of SARS-CoV-2-specific antibodies. The basic principle of convalescent plasma therapy for COVID-19 patients is that this treatment provides specific antibodies from a recovered host to an affected individual. This strategy has a long history of success in other viral respiratory diseases [67]. A recent systematic study by Huang et al. revealed that antibody-mediated immunity may affect the dynamics of SARS-CoV-2-specific antibodies in protecting patients from viral reinfection [68]. In a study conducted by Shen et al., five critically ill patients with severe COVID-19 were treated with convalescent plasma therapy [69]. After plasma infusion, the NAb titer was significantly increased, leading to a decrease in the viral load and an improvement in patient clinical condition within a few days. Thus, the specific antibodies in the plasma during convalescence may be helpful in eliminating SARS-CoV-2 and improving symptoms. Although antibody-mediated immunotherapy may have some benefits, randomized controlled trials are urgently needed to determine its efficacy and safety [70].

### Vaccine development based on enhancing SARS-COV-2-specific humoral immunity

The S protein of coronaviruses is commonly used in coronavirus vaccine design because it plays a crucial role in mediating viral entry into host cells [71]. The RBD of the viral S protein contains multiple dominant neutralizing epitopes and can be used as an essential antigen in the development of COVID-19 vaccines (Fig. 1). Previous studies have revealed that the Fc fragment of human IgG in the RBD-based vaccine RBD-Fc can be used as a vital immunopotentiator to enhance RBD immunogenicity [72, 73]. In a recent study, a candidate subunit vaccine comprising the SARS-CoV-2 RBD and human IgG Fc fragment was used as an immunopotentiator to immunize mice and produced a high titer of RBD-specific antibodies with vigorous neutralizing activity against pseudotyped and authentic SARS-CoV-2 infections, indicating that the RBD-Fc vaccine can induce relatively long-term NAb responses [74]. Moreover, another recent study reported that NAbs induced by DNA and Ad26-based vaccines are closely related to a decrease in the viral load in rhesus monkeys [75]. Collectively, these studies suggest that NAb titers serve as useful indicators for evaluating SARS-CoV-2 vaccines in both preclinical and clinical studies. However, these correlations need to be further confirmed in a large human population [76]. Some vaccines developed for SARS-CoV-2 infection may not be able to completely prevent SARS-CoV-2 infection because the S protein of SARS-CoV-2 has undergone significant mutations [77, 78]. The development of a safe and effective COVID-19 vaccine that induces high NAb titers and prevents SARS-CoV-2 reinfection has become the key in controlling the COVID-19 pandemic globally. During the ongoing COVID-19 pandemic, many candidate vaccines have been rapidly tested in clinical trials, some of which have even been used for vaccinating the general population. Although some of these candidate vaccines have shown good prospects in inducing NAb and T cell responses, whether these vaccines protect immunized individuals from SARS-CoV-2 reinfection, particularly from infection with SARS-CoV-2 with significant mutations, remains unknown [74].

The potential risk of vaccine-induced ADE, which is a potential obstacle to antibody-based vaccines and therapies and could increase the severity of COVID-19, should not be ignored. ADE is a mechanism through which the pathogenesis of certain viral infections is enhanced in the presence of subneutralizing or cross-reactive nonneutralizing antiviral antibodies [79]. Previous studies have revealed that after macrophages are infected with dengue virus or feline infectious peritonitis virus, ADE enhances viral replication [80, 81]. Regarding SARS-CoV and MERS-CoV infections, the presence of subneutralizing or cross-reactive non-NAbs has the theoretical potential to enhance infection and trigger harmful immunopathology [82, 83]. Furthermore, ADE can increase the severity of multiple viral infections, including those caused by other respiratory viruses, such as respiratory syncytial virus and measles virus [84, 85]. However, suggestions regarding ADE in COVID-19 have garnered justifiable criticism owing to limited evidence and the lack of a robust demonstration in animal models, as in vitro evidence for coronaviruses is not indicative of disease pathology in the absence of ongoing and comprehensive innate and adaptive immunity in the dish [86].

## Conclusions and perspectives

In this review, we summarized the recent progress in experimental detection methods and the understanding of the characteristics and dynamics of specific humoral immunity against SARS-CoV-2 in COVID-19 patients and those immunized with a SARS-CoV-2 vaccine. There is an urgent need to confirm the correlations between antibody (especially NAb) titers and protection against SARS-CoV-2 reinfection in future studies. However, the emergence of cross-reactions among different coronavirus antigens in the development of screening technology and the risk of ADE in COVID-19 vaccination should not be ignored.

## References

[1] Phelan AL, Katz R, Gostin LO (2020). The novel coronavirus originating in Wuhan, China: challenges for global health governance. JAMA.

[2] Zhou P, Yang XL, Wang XG, Hu B, Zhang L, Zhang W (2020). Addendum: a pneumonia outbreak associated with a new coronavirus of probable bat origin. Nature.

[3] Zhang YZ, Holmes EC (2020). A genomic perspective on the origin and emergence of SARS-CoV-2. Cell.

[4] Walls AC, Park YJ, Tortorici MA, Wall A, McGuire AT, Veesler D (2020). Structure, function, and antigenicity of the SARS-CoV-2 spike glycoprotein. Cell.

[5] Wrapp D, Wang N, Corbett KS, Goldsmith JA, Hsieh CL, Abiona O (2020). Cryo-EM structure of the 2019-nCoV spike in the prefusion conformation. Science.

[6] Dorner T, Radbruch A (2007). Antibodies and B cell memory in viral immunity. Immunity.

[7] Palm AKE, Henry C (2019). Remembrance of things past: long-term B cell memory after infection and vaccination. Front Immunol.

[8] Guo L, Ren L, Yang S, Xiao M, Chang, Yang F (2020). Profiling early humoral response to diagnose novel coronavirus disease (COVID-19). Clin Infect Dis.

[9] Wu F, Zhao S, Yu B, Chen YM, Wang W, Song ZG (2020). A new coronavirus associated with human respiratory disease in China. Nature.

[10] Petherick A (2020). Developing antibody tests for SARS-CoV-2. Lancet.

[11] van der Hoek L, Pyrc K, Berkhout B (2006). Human coronavirus NL63, a new respiratory virus. FEMS Microbiol Rev.

[12] Lou B, Li TD, Zheng SF, Su YY, Li ZY, Liu W (2020). Serology characteristics of SARS-CoV-2 infection after exposure and post-symptom onset. Eur Respir J.

[13] Cai XF, Chen J, Li Hu J, Long QX, Deng HJ, Liu P (2020). A peptide-based magnetic chemiluminescence enzyme immunoassay for serological diagnosis of coronavirus disease 2019. J Infect Dis.

[14] Whitman JD, Hiatt J, Mowery CT, Shy BR, Yu R, Yamamoto TN (2020). Evaluation of SARS-CoV-2 serology assays reveals a range of test performance. Nat Biotechnol.

[15] Cervia C, Nilsson J, Zurbuchen Y, Valaperti A, Schreiner J, Wolfensberger A (2021). Systemic and mucosal antibody responses specific to SARS-CoV-2 during mild versus severe COVID-19. J Allergy Clin Immunol.

[16] Gaebler C, Wang Z, Lorenzi J, Muecksch F, Finkin S, Tokuyama M (2021). Evolution of antibody immunity to SARS-CoV-2. Nature.

[17] Orologas-Stavrou N, Politou M, Rousakis P, Kostopoulos IV, Ntanasis-Stathopoulos I, Jahaj E (2020). Peripheral blood immune profiling of convalescent plasma donors reveals alterations in specific immune subpopulations even at 2 months post SARS-CoV-2 infection. Viruses.

[18] Zhang LX, Miao SY, Qin ZH, Wu JP, Chen HY, Sun HB (2020). Preliminary analysis of B- and T-cell responses to SARS-CoV-2. Mol Diagn Ther.

[19] Jin Y, Wang M, Zuo Z, Fan C, Ye F, Cai Z (2020). Diagnostic value and dynamic variance of serum antibody in coronavirus disease 2019. Int J Infect Dis.

[20] Xiang F, Wang X, He X, Peng Z, Yang B, Zhang J (2020). Antibody detection and dynamic characteristics in patients with coronavirus disease 2019. Clin Infect Dis.

[21] Ma H, Zeng W, He H, Zhao D, Jiang D, Zhou P (2020). Serum IgA, IgM, and IgG responses in COVID-19. Cell Mol Immunol.

[22] Roltgen K, Boyd SD (2021). Antibody and B cell responses to SARS-CoV-2 infection and vaccination. Cell Host Microbe.

[23] Tang J, Ravichandran S, Lee Y, Grubbs G, Coyle EM, Klenow L (2021). Antibody affinity maturation and plasma IgA associate with clinical outcome in hospitalized COVID-19 patients. Nat Commun.

[24] Chen Y, Zuiani A, Fischinger S, Mullur J, Atyeo C, Travers M (2020). Quick COVID-19 Healers sustain anti-SARS-CoV-2 antibody production. Cell.

[25] Wang Y, Li J, Li H, Lei P, Shen G, Yang C (2021). Persistence of SARS-CoV-2-specific antibodies in COVID-19 patients. Int Immunopharmacol.

[26] Zohar T, Loos C, Fischinger S, Atyeo C, Wang C, Slein MD (2020). Compromised humoral functional evolution tracks with SARS-CoV-2 mortality. Cell.

[27] Hansen CB, Jarlhelt I, Pérez-Alós L, Hummelshøj Landsy L, Loftager M, Rosbjerg A (2021). SARS-CoV-2 antibody responses are correlated to disease severity in COVID-19 convalescent individuals. J Immunol.

[28] Qu J, Wu C, Li X, Zhang G, Jiang Z, Li X (2020). Profile of immunoglobulin G and IgM antibodies against severe acute respiratory syndrome coronavirus 2 (SARS-CoV-2). Clin Infect Dis.

[29] Zhao J, Yuan Q, Wang H, Liu W, Liao X, Su Y (2020). Antibody responses to SARS-CoV-2 in patients of novel coronavirus disease 2019. Clin Infect Dis.

[30] Long QX, Liu BZ, Deng HJ, Wu GC, Deng K, Chen YK (2020). Antibody responses to SARS-CoV-2 in patients with COVID-19. Nat Med.

[31] Lynch KL, Whitman JD, Lacanienta NP, Beckerdite EW, Kastner SA, Shy BR (2021). Magnitude and kinetics of anti-severe acute respiratory syndrome coronavirus 2 antibody responses and their relationship to disease severity. Clin Infect Dis.

[32] Lin Y, Wu C, Wang X, Liu S, Zhao K, Kemper T (2020). Glucosamine promotes hepatitis B virus replication through its dual effects in suppressing autophagic degradation and inhibiting MTORC1 signaling. Autophagy.

[33] Lynch KL, Whitman JD, Lacanienta NP, Beckerdite EW, Kastner SA, Shy BR (2020). Magnitude and kinetics of anti-SARS-CoV-2 antibody responses and their relationship to disease severity. Clin Infect Dis.

[34] Wang Y, Zhang L, Sang L, Ye F, Ruan S, Zhong B (2020). Kinetics of viral load and antibody response in relation to COVID-19 severity. J Clin Investig.

[35] Casadevall A, Joyner MJ, Pirofski LA (2020). SARS-CoV-2 viral load and antibody responses: the case for convalescent plasma therapy. J Clin Investig.

[36] Yu HQ, Sun BQ, Fang ZF, Zhao JC, Liu XY, Li YM (2020). Distinct features of SARS-CoV-2-specific IgA response in COVID-19 patients. Eur Respir J.

[37] Long QX, Tang XJ, Shi QL, Li Q, Deng HJ, Yuan J (2020). Clinical and immunological assessment of asymptomatic SARS-CoV-2 infections. Nat Med.

[38] Long QX, Jia YJ, Wang X, Deng HJ, Cao XX, Yuan J (2021). Immune memory in convalescent patients with asymptomatic or mild COVID-19. Cell Discov.

[39] Mazzoni A, Maggi L, Capone M, Spinicci M, Salvati L, Colao MG (2020). Cell-mediated and humoral adaptive immune responses to SARS-CoV-2 are lower in asymptomatic than symptomatic COVID-19 patients. Eur J Immunol.

[40] Schöler L, Le-Trilling VTK, Eilbrecht M, Mennerich D, Anastasiou OE, Krawczyk A (2020). A novel in-cell ELISA assay allows rapid and automated quantification of SARS-CoV-2 to analyze neutralizing antibodies and antiviral compounds. Front Immunol.

[41] Choe PG, Kang CK, Suh HJ, Jung J, Song KH, Bang JH (2021). Waning antibody responses in asymptomatic and symptomatic SARS-CoV-2 infection. Emerg Infect Dis.

[42] Zhang Y, Xu J, Jia R, Yi C, Gu W, Liu P (2020). Protective humoral immunity in SARS-CoV-2 infected pediatric patients. Cell Mol Immunol.

[43] Wu H, Zhu H, Yuan C, Yao C, Luo W, Shen X (2020). Clinical and immune features of hospitalized pediatric patients with coronavirus disease 2019 (COVID-19) in Wuhan, China. JAMA Netw Open.

[44] Felsenstein S, Hedrich CM (2020). SARS-CoV-2 infections in children and young people. Clin Immunol.

[45] Pierce CA, Preston-Hurlburt P, Dai Y, Aschner CB, Cheshenko N, Galen B (2020). Immune responses to SARS-CoV-2 infection in hospitalized pediatric and adult patients. Sci Transl Med.

[46] Corman VM, Landt O, Kaiser M, Molenkamp R, Meijer A, Chu DK (2020). Detection of 2019 novel coronavirus (2019-nCoV) by real-time RT-PCR. Eurosurveillance.

[47] Udugama B, Kadhiresan P, Kozlowski HN, Malekjahani A, Osborne M, Li V (2020). Diagnosing COVID-19: the disease and tools for detection. ACS Nano.

[48] Krammer F, Simon V (2020). Serology assays to manage COVID-19. Science.

[49] Lerner AM, Eisinger RW, Lowy DR, Petersen LR, Humes R, Hepburn M (2020). The COVID-19 serology studies workshop: recommendations and challenges. Immunity.

[50] Shi Q, Hu Y, Peng B, Tang XJ, Wang W, Su K (2021). Effective control of SARS-CoV-2 transmission in Wanzhou. China Nat Med.

[51] Gudbjartsson DF, Norddahl GL, Melsted P, Gunnarsdottir K, Holm H, Eythorsson E (2020). Humoral immune response to SARS-CoV-2 in Iceland. N Engl J Med.

[52] Stringhini S, Wisniak A, Piumatti G, Azman AS, Lauer SA, Baysson H (2020). Seroprevalence of anti-SARS-CoV-2 IgG antibodies in Geneva, Switzerland (SEROCoV-POP): a population-based study. Lancet.

[53] Pollán M, Pérez-Gómez B, Pastor-Barriuso R, Oteo J, Hernán MA, Pérez-Olmeda M (2020). Prevalence of SARS-CoV-2 in Spain (ENE-COVID): a nationwide, population-based seroepidemiological study. Lancet.

[54] Kreer C, Zehner M, Weber T, Ercanoglu MS, Gieselmann L, Rohde C (2020). Longitudinal isolation of potent near-germline SARS-CoV-2-neutralizing antibodies from COVID-19 patients (vol 182, pg 843, 2020). Cell.

[55] Ju B, Zhang Q, Ge J, Wang R, Sun J, Ge X (2020). Human neutralizing antibodies elicited by SARS-CoV-2 infection. Nature.

[56] Wang C, Li W, Drabek D, Okba N, van Haperen R, Osterhaus A (2020). A human monoclonal antibody blocking SARS-CoV-2 infection. Nat Commun.

[57] Zost SJ, Gilchuk P, Case JB, Binshtein E (2020). Potently neutralizing and protective human antibodies against SARS-CoV-2. Nature.

[58] Chi X, Yan R, Zhang J, Zhang G, Zhang Y, Hao M (2020). A neutralizing human antibody binds to the N-terminal domain of the Spike protein of SARS-CoV-2. Science.

[59] Zhu FC, Guan XH, Li YH, Huang JY, Jiang T, Hou LH (2020). Immunogenicity and safety of a recombinant adenovirus type-5-vectored COVID-19 vaccine in healthy adults aged 18 years or older: a randomised, double-blind, placebo-controlled, phase 2 trial. Lancet.

[60] Wang K, Long QX, Deng HJ, Hu J, Gao QZ, Zhang GJ (2021). Longitudinal dynamics of the neutralizing antibody response to SARS-CoV-2 infection. Clin Infect Dis.

[61] Du L, Zhao G, Lin Y, Chan C, He Y, Jiang S (2008). Priming with rAAV encoding RBD of SARS-CoV S protein and boosting with RBD-specific peptides for T cell epitopes elevated humoral and cellular immune responses against SARS-CoV infection. Vaccine.

[62] Fett C, DeDiego ML, Regla-Nava JA, Enjuanes L, Perlman S (2013). Complete protection against severe acute respiratory syndrome coronavirus-mediated lethal respiratory disease in aged mice by immunization with a mouse-adapted virus lacking E protein. J Virol.

[63] Chandrashekar A, Liu J, Martinot AJ, McMahan K, Mercado NB, Peter L (2020). SARS-CoV-2 infection protects against rechallenge in rhesus macaques. Science.

[64] Deng W, Bao L, Liu J, Xiao C, Liu J, Xue J (2020). Primary exposure to SARS-CoV-2 protects against reinfection in rhesus macaques. Science.

[65] Röltgen K, Powell AE, Wirz OF, Stevens BA, Hogan CA, Najeeb J (2020). Defining the features and duration of antibody responses to SARS-CoV-2 infection associated with disease severity and outcome. Sci Immunol.

[66] Seow J, Graham C, Merrick B, Acors S, Pickering S, Steel K (2020). Longitudinal observation and decline of neutralizing antibody responses in the three months following SARS-CoV-2 infection in humans. Nat Microbiol.

[67] Bloch EM, Shoham S, Casadevall A, Sachais BS, Shaz B, Winters JL (2020). Deployment of convalescent plasma for the prevention and treatment of COVID-19. J Clin Investig.

[68] Huang AT, Garcia-Carreras B, Hitchings M, Yang B, Katzelnick LC, Rattigan SM (2020). A systematic review of antibody mediated immunity to coronaviruses: kinetics, correlates of protection, and association with severity. Nat Commun.

[69] Shen C, Wang Z, Zhao F, Yang Y, Li J, Yuan J (2020). Treatment of 5 critically ill patients with COVID-19 with convalescent plasma. JAMA.

[70] Wooding DJ, Bach H (2020). Treatment of COVID-19 with convalescent plasma: lessons from past coronavirus outbreaks. Clin Microbiol Infect.

[71] Song Z, Xu Y, Bao L, Zhang L, Yu P, Qu Y (2019). From SARS to MERS, thrusting coronaviruses into the spotlight. Viruses.

[72] Du L, Zhao G, He Y, Guo Y, Zheng BJ, Jiang S (2007). Receptor-binding domain of SARS-CoV spike protein induces long-term protective immunity in an animal model. Vaccine.

[73] He Y, Li J, Li W, Lustigman S, Farzan M, Jiang S (2006). Cross-neutralization of human and palm civet severe acute respiratory syndrome coronaviruses by antibodies targeting the receptor-binding domain of spike protein. J Immunol.

[74] Liu Z, Xu W, Xia S, Gu C, Wang X, Wang Q (2020). RBD-Fc-based COVID-19 vaccine candidate induces highly potent SARS-CoV-2 neutralizing antibody response. Signal Transduct Target Ther.

[75] Mercado NB, Zahn R, Wegmann F, Loos C, Chandrashekar A, Yu J (2020). Single-shot Ad26 vaccine protects against SARS-CoV-2 in rhesus macaques. Nature.

[76] Dagotto G, Yu JY, Barouch DH (2020). Approaches and challenges in SARS-CoV-2 vaccine development. Cell Host Microbe.

[77] Garcia-Beltran WF, Lam EC, St Denis K, Nitido AD, Garcia ZH, Hauser BM (2021). Multiple SARS-CoV-2 variants escape neutralization by vaccine-induced humoral immunity. Cell.

[78] Planas D, Bruel T, Grzelak L, Guivel-Benhassine F, Staropoli I, Porrot F (2021). Sensitivity of infectious SARS-CoV-2 B.1.1.7 and B.1.351 variants to neutralizing antibodies. Nat Med.

[79] Bournazos S, Gupta A, Ravetch JV (2020). The role of IgG Fc receptors in antibody-dependent enhancement. Nat Rev Immunol.

[80] Dejnirattisai W, Jumnainsong A, Onsirisakul N, Fitton P, Vasanawathana S, Limpitikul W (2010). Cross-reacting antibodies enhance dengue virus infection in humans. Science.

[81] Sridhar S, Luedtke A, Langevin E, Zhu M, Bonaparte M, Machabert T (2018). Effect of dengue serostatus on dengue vaccine safety and efficacy. N Engl J Med.

[82] Tetro JA (2020). Is COVID-19 receiving ADE from other coronaviruses?. Microbes Infect.

[83] Wan Y, Shang J, Sun S, Tai W, Chen J, Geng Q (2020). Molecular mechanism for antibody-dependent enhancement of coronavirus entry. J Virol.

[84] Graham BS (2016). Vaccines against respiratory syncytial virus: the time has finally come. Vaccine.

[85] Polack FP (2007). Atypical measles and enhanced respiratory syncytial virus disease (ERD) made simple. Pediatr Res.

[86] Sharma A (2020). It is too soon to attribute ADE to COVID-19. Microbes Infect.

